# Validating the Balanced Inventory of Desirable Reporting in a low literacy adolescent population in Burkina Faso

**DOI:** 10.1038/s41598-025-23145-1

**Published:** 2025-11-10

**Authors:** Karolin Kirchgaesser, Till Bärnighausen, Mamadou Bountogo, Ali Sié, Guy Harling

**Affiliations:** 1 Heidelberg Institute of Global Health (HIGH), University of Heidelberg, Heidelberg, Germany; 2 Africa Health Research Institute (AHRI), KwaZulu-Natal, South Africa; 3 Department of Global Health and Population, Harvard T.H. Chan School of Public Health, Boston, MA USA; 4 Institute for Global Health, University College London, Mortimer Market Centre, London, WC1E 6JB UK; 5 Centre de Recherche en Santé de Nouna, Nouna, Burkina Faso; 6 School of Nursing and Public Health, University of KwaZulu-Natal, Durban, South Africa; 7 MRC/WITS Rural Public Health and Health Transitions Research Unit (Agincourt), Faculty of Health Sciences, University of the Witwatersrand, Johannesburg, South Africa

**Keywords:** Social desirability, Burkina faso, Adolescence, Validation, Human behaviour, Public health

## Abstract

Socially desirable responses to survey questions may be universal, but scales to capture the phenomenon are unvalidated in low-education and resource-limited settings. We therefore conducted a validation of the 16-item Balanced Inventory of Desirable Reporting (BIDR) short form in a two-round health survey of 1291 12–20 year-olds in rural Burkina Faso in 2017 and 2018. Confirmatory factor analysis (CFA) of the scale’s designed dimensionality found poor fit (CFI = 0.50, TLI = 0.42, RMSEA = 0.10, SRMR = 0.08). Exploratory factor analysis of Wave 1 data suggested a novel 11-item, 2-factor structure, with all but two of the original scale’s Self Deceptive Enhancement items discarded. CFA in Wave 2 using this novel structure gave poor fit indices (CFI = 0.62, TLI = 0.51, RMSEA = 0.10, SRMR = 0.07), test-retest reliability was low (ICC(A,1) = 0.06, Pearson’s *r* = 0.06, R^2^ = 0.004) and internal consistency was unsatisfactory (α and ω < 0.70) across waves for both scales. Measurement invariance was confirmed for age but not gender. This failure of BIDR implementation may reflect issues with item translation and delivery, locally appropriate content or use of reverse-coding in a low-education sample. It is possible, but less likely, that it reflects non-universality of the SDR construct. Our work highlights the importance of validating instruments in new study populations.

**Supplementary Information:**

The online version contains supplementary material available at 10.1038/s41598-025-23145-1.

## Introduction

Socially Desirable Responding (SDR) describes the phenomenon of individuals giving overly positive self-descriptions, that is “over-reporting positive behavior or under-reporting negative behavior”^1^. In this context, “over-reporting” expresses a central “departure from reality” which distinguishes SDR from content dimensions of personality^2^. SDR may reflect unintentional deception of oneself or intentional deception of others. Unintentional deception is commonly described as Self Deceptive Enhancement (SDE) and predicts “overconfidence, hindsight and overclaiming”^1^. Intentional deception, i.e., aiming to portray oneself in a favourable light, is often called Impression Management (IM)^3^; IM varies by context, notably being higher in public than in private settings. Individuals’ levels of SDR are believed to be a function of both personal and social factors. At the individual psychological level, SDE is correlated with the Big Five personality traits of Extraversion and Openness, while IM is associated with Agreeableness and Conscientiousness^2^.

*SDR and adolescent health.* SDR has been interpreted both as a substantive character trait, and a response style and thus noise. Insofar as SDR is not a trait of substantive interest to the researcher, it is an almost-unavoidable source of bias in self-response questionnaires that are required to capture non-observable phenomena. In the context of health research these phenomena include knowledge, attitudes, beliefs and private practices such as sexual behaviour and illicit substance use, all of which are subject to social or legal sanction. As a result, concern about SDR is common in the field.

SDR is of particular relevance for adolescent health research, since key health outcomes for this group – sexual behaviour, substance use, reporting of harassment and poor mental health – are sensitive and thus likely to suffer from SDR. Most adolescent SDR research has been conducted among college students in higher-income, English-speaking countries^19–21^. However, the fastest growing population of adolescents globally lives in lower-literacy and lower-media-exposed settings in lower- and middle-income countries^22^.

*Reducing SDR.* One approach to concern about SDR bias is to try and decrease it (particularly IM) through adjustments to instruments, item wording or interview settings. Face-to-face interviews can provoke SDR if the respondent suspects potential violation of privacy, particularly when asking sensitive questions^4,5^. To avoid this, several methods that increase respondent privacy and anonymity have been tried, including: respondent-led self-interviews with or without audio-recorded questions^5,6^; nonverbal response cards^7,8^; ballot-boxes^4^; list randomization^9^; and the random response method^10^. These methods have limitations, particularly for respondents with low linguistic and computer literacy or little experience of abstract conceptualization^11,12^. Direct evidence for these methods’ impact on SDR is limited, although they frequently lead to greater reporting of sensitive, socially undesirable responses^5,6^.

*Measuring SDR*. Alternatively, SDR may be measured, at which point it can be assessed or potentially controlled when analysing other outcomes^13,14^. Such control presumes SDR to be a response style rather than of substantive import, is complex if SDR lies on the causal pathway between exposures and outcomes of interest^11^, and may affect variance validity and thus decrease survey validity and thus potentially affect survey-elicited health research^13,15^. Nevertheless, several measures have been developed to quantify SDR. Possibly the first was the one-dimensional Edwards Social Desirability Scale, using 39 items from the Minnesota Multiphasic Personality Inventory which are either very desirable or very undesirable^16^. Shortly afterwards the Marlowe-Crowne Social Desirability Scale (MCSDS) was developed using 33 items which present either undesirable but common or desirable yet uncommon everyday life behaviours, rather than psychopathological ones^17^. Subsequent use suggested that the MCSDS loads more heavily on IM and captures something closer to a trait of need for approval^13^. Lastly, the Balanced Inventory of Desirable Responding (BIDR) explicitly identified two dimensions of SDR – IM and SDE^3^. The inventory is based on Sackeim and Gur’s Self- and Other-Deception Questionnaires but with self-deception focused on self-enhancement rather than self-defence^18^.

SDR-capturing scales are predicated on a stable concept of what is socially desirable; If the construct or direction of social desirability in reporting is not universal we may not be able to use a single scale worldwide^23,24^. It has been argued that SDE is closely aligned to or reflects Agency – “getting ahead”, the key characteristic of an individualistic society – while IM reflects Communion – “getting along”, the key characteristic of collectivist societies. If this is the case, then agency and communion may be more useful ways to conceptualize SDR on a content level^15^. At the group level more collectivist societies, where conformity, in-group harmony and self-control are desirable and face-keeping thus plays a major role, are expected to promote greater impression management^25^. Past work has suggested that SDR scales have nomological validity and structural invariance (internal consistency and two-factor form) across cultural settings: USA and Singapore^26^; eight African countries, including Burkina Faso, and Switzerland^23^; 26 countries in Europe, North America and Asia^21^. However, distributions differed between societies, with higher IM levels in more collectivist settings^27^ despite personal individualism being unrelated to IM^26^. Evidence therefore suggests that SDR may well be present worldwide, but scalar equivalence cannot be assumed, and may vary subject to cultural differences^25^.

*Study objective.* Given the limited evidence available on SDR we aimed to analyse the validity and reliability of a low-burden SDR scale in a semi-rural, low literacy setting. Rural Burkina Faso represents a setting with a high proportion of adolescents^28^, low literacy (50% among 15–24 year olds^28^) and low media exposure^29^. It also represents a setting where health data is commonly captured face-to-face in ways that maximize the risk of SDR^30^. We therefore assessed SDR in a cohort of over 1000 adolescents in Nouna department in northwestern Burkina Faso.

## Methods

### Study site

We used data from two rounds of data collected on a cohort of adolescents (ages 12–19 at baseline) in the Nouna Health and Demographic Surveillance System (HDSS), run by the Centre de Recherche en Santé de Nouna (CRSN) and covering Nouna town and 58 surrounding villages (total population ~ 107,000 in 2015) in the Boucle de Mouhoun region of western Burkina Faso^31^. The cohort was part of a wider ARISE Network Adolescent Health study, a collaboration between research institutions in seven African countries alongside US and European colleagues^32^.

### Sample and study procedures

The cohort was chosen using a two-step stratified sampling process from a 2015 HDSS census. First, 10 villages within the census area were selected to capture the five main ethnicities present in the area, and thus the breadth of religious affiliation. From all census children in these villages projected to be aged 12–19 years on 1 October 2017, a sample of 1795 was drawn. Second, a sample of 749 age-eligible children was drawn from one of Nouna town’s randomly chosen seven sectors, to provide an urban/rural-ratio in the sample in line with the HDSS population as a whole.

The cohort study collected self-report information on socio-demographics, behaviours, health practices and health outcomes. Interviews were conducted by trained fieldworkers using tablet computers, either in French or translated into the local languages (mostly Dioula). These local languages are rarely written – even by those literate in French – limiting the potential of standard translation back-translation procedures^33^. Instead, translation practice was an integral part of fieldworker training. Uncertainties regarding the meaning of specific items were discussed in a group setting during training. Fieldworkers could also address questions individually. Individuals were approached at their homes in November and December 2017, and for those who participated for a second time in November and December 2018.

### Ethics

The study was approved by the Institutional Ethics Committee of the CRSN (reference number: 2017-08). The Ethics Committee of the University of Heidelberg’s Medical Faculty exempted the study from review, since all data provided to non-CRSN staff was anonymised. Written informed consent was obtained from all participants aged 18 and older; younger participants provided written informed assent and written informed parent/guardian consent. A literate witness assisted with informed consent in case of illiteracy. All research was conducted in accordance with the Declaration of Helsinki.

### Outcome measure

The BIDR-16 is a short form of Paulhus’ original 40-item scale^3,34^ which asks eight questions relating to each of the two original factors IM and SDE, half reverse-coded to avoid straight-line response patterns (precise wording in English and French provided in Supplementary Table S4)^1^. Each question is scored on a 7-point Likert scale (“strongly disagree” to “strongly agree”) and treated as continuous^35^. We summed responses to all items to generate a factor-specific continuous (potential range 8–56) and an overall score (potential range 16–112), with higher scores indicating higher SDR^36,37^.

### Other measures

We created several variables based on existing literature and available constructs within the ARISE questionnaire that capture (un)desirable or locally normative behaviours or reflect perceptions of self-esteem.

We grouped respondents’ age and gender in an 8-category age-gender variable: ages 12–13, 14–15, 16–17, 18–21 for male and female. Education was measured in two ways: currently in school (yes/no) and the highest level of education attained (None; Primary; Post-primary; Secondary or higher). For life satisfaction we used the Students’ Life Satisfaction Scale (SLSS), a 7-item, 6-point Likert scale (scoring: sum, range: 7–42) (detailed wording for SLSS questions is provided in Supplementary Table S5). Handwashing frequency before eating over the past 30 days was recorded using a 5-point Likert scale and then dichotomized into binary scoring (always/not always). We measured Sexual Violence by inquiring about any lifetime experience of: verbal harassment; unwanted sexual touching; attempted rape; rape (scoring: sum, range 0–4). Alcohol Use is indicated by days drunk alcohol in the past month (scoring: binary: ≥1 drink in past month vs. one or none). To assess respondents’ nutritional status, the mid-upper arm circumference of the dominant arm was measured by fieldworkers using a measuring tape (scoring: continuous (cm) average of two measurements). Diet is indicated by any consumption of: green leafy vegetables; other vegetables; legumes; dairy in the past 24 hours (scoring: sum, range 0–4).

### Analytic plan

All analyses were conducted in R Statistical software (v4.3.3; R Core Team 2024). We first calculated descriptive statistics for all variables using proportions or mean, standard deviation and median as appropriate. To determine sampling adequacy we conducted Kaiser-Meyer-Olkin (KMO)^38^ and Bartlett’s sphericity tests^39^ of all BIDR items for both waves respectively. BIDR missing values were handled using pairwise deletion. For predictor variables we used casewise deletion after verifying that not more than 6% were missing (Supplementary Table S1). BIDR reverse-coded items were recoded. We follow the approach by Boateng et al.^35^ for the validation process.

### Dimensionality

We first conducted confirmatory factor analysis (CFA) for each wave to assess whether items loaded onto the original two factors (IM and SDE). We assessed model fit using four indices with specific cut-off values – standardised root mean square residual (SRMR, < 0.08); root mean square error of approximation (RMSEA, < 0.05); comparative fit index (CFI, > 0.93); Tucker-Lewis Index (TLI > 0.95) – and compared the observed and theoretically proposed covariance matrices using a $\:{{\upchi\:}}^{2}$ test. We used modification indices to determine whether covariances between certain items would lead to a χ^2^ reduction, i.e., improved model fit.

In this first CFA we found that initial model fit was unsatisfactory, meaning our data did not confirm the original two-factor structure of the BIDR. Consequently, we performed exploratory factor analysis (EFA) on Wave 1 data^40^ using oblique rotation, i.e., allowing items to correlate, to try and identify a valid factor structure for our data. We retained factors based on a scree plot and parallel analysis. We next dropped BIDR items with loadings of < 0.3 or cross loading, i.e., absolute loading difference between items <. 1, re-running EFA after each item deletion. To validate the new structure we then ran a second CFA on the Wave 2 sample.

Finally, we assessed measurement invariance by splitting our sample by age, by gender (12–15 vs. 16–21; male and female) and by the two largest interview language groups (French and Dioula). Configural invariance was established as a baseline model by assessing overall model fit. Metric invariance was assessed by comparing the metric model (constrained loadings) with the configural model (no constraints) using a $\:{\chi\:}^{2}$ difference test. Scalar invariance, i.e. invariant intercepts, was confirmed by comparing the metric model with a third model that additionally constrains intercepts to be equal^41^. We followed the reporting guidelines of Putnick et al.^42^.

### Reliability

To assess internal consistency we calculated McDonald’s omega and Cronbach’s alpha for each wave and subscale for the original scale and our final preferred scale, regarding each subscale as a latent construct. We considered coefficients ≥ 0.7 as acceptable and ≥ 0.8 as preferred^43^. The BIDR was administered to the same group of respondents one year apart. To assess test-retest reliability we calculated two-way mixed effect, single measure, absolute agreement Intraclass Correlation Coefficients as an assessment of agreement of measurements between the two points in time for our preferred scale^44^. We additionally calculated Pearson’s correlation coefficient for each year’s BIDR-sum scores and regressed Wave 2 sum score on Wave 1 sum score.

### Validity

We evaluated the scale’s convergent and discriminant construct validity using linear regression of respondent’s summed BIDR score overall and for each subscale on the relevant variable. We adjusted for age and gender in all models and for nonverbal response card^8^ trial arm for variables collected in that survey section^41^. Convergent validity coefficients are expected to be statistically significant; divergent validity coefficients non-significant. We hypothesized positive correlations between BIDR and desirable measures, specifically: life satisfaction^45^; compliance with normative hygienic handwashing customs^11^; and confidence in one’s own judgement. We hypothesized negative associations between BIDR and: formal educational attainment – noting mixed past evidence^46–48^; stigmatized outcomes such as substance use and sexual violence^49–51^. For divergent validity, we assumed that respondents’ dietary patterns and nutritional status would be distinct from SDR.

## Results

Of 2544 sampled adolescents, 2271 remained eligible and resident in 2017, 1693 (75%) were contactable and after 39 non-consents (3%), 1644 individuals completed the Wave 1 interview. In 2018, 1366 (83%) of Wave 1 participants were still living in the area, 72 (5.3%) did not consent to re-participate and 3 others were unable to consent, leaving 1291 (79% of Wave 1 respondents) who were re-interviewed and included here. The sample was more male than female and skewed towards younger age in 2017. More than half had not continued past primary education (Table 1). KMO and Bartlett’s test of sphericity suggested sampling adequacy for factor analysis (Wave 1: KMO = 0.71, Bartlett’s *p* < 0.001, Wave 2: KMO = 0.65, Bartlett’s *p* < 0.001).

**Table 1:** Descriptive characteristics.

	Wave 1		Wave 2	
	n	% or Mean (SD)	n	% or Mean (SD)
Male	1291	59.0	1291	58.9
Age	1291		1291	
12–13 female		14.2		5.8
14–15 female		10.8		14.6
16–17 female		8.2		8.2
18–21 female		7.7		12.5
12–13 male		19.4		9.1
14–15 male		14.9		17.4
16–17 male		13.2		14.3
18–21 male		11.5		18.3
Interview language			1291	
French			593	45.9
Dioula			590	45.7
Mooré			34	2.6
Dafin			62	4.8
Bwamu			11	0.9
Marka			1	0.1
Currently in school^1^	1291	54.5	1291	52.0
Highest school level	1291		1291	
None or Koranic school		24.4		27.5
Primary		39.7		30.4
Post Primary		33.2		38.0
Secondary or higher		2.7		4.1
SLSS	1278	21.9 (6.2)	1285	22.0 (5.0)
Handwashing	1291	1.97 (0.17)	1291	1.92 (0.26)
Sexual violence	1226	0.38 (0.83)	1259	0.24 (0.68)
Alcohol use	1287	1.06 (0.26)	1274	1.07 (0.26)
Confidence			1281	3.32 (0.75)
Nutritional status (MUAC)	1287	22.9 (3.65)	1291	24.6 (3.39)
Diet	1284	1.71 (1.09)	1280	1.43 (1.03)
BIDR overall	1250	76.0 (13.9)	1269	74.0 (12.1)
BIDR IM	1278	38.8 (9.9)	1287	37.2 (8.2)
BIDR SDE	1257	37.2 (7.5)	1272	36.8 (6.9)

^1^Including Koranic school, BIDR: balanced inventory of desirable responding with its two original subscales IM: impression management and SDE: self-deceptive enhancement, SLSS: students’ life satisfaction scale, MUAC: mid-upper arm circumference, SD: standard deviation.

### Dimensionality

*Confirmatory factor analysis*. Conducting 2-factor CFA we found in both waves that the exact and incremental (CFI, TLI) fit indices were poor, while absolute fit indices (RMSEA, SRMR) were more acceptable (Table 2). When we allowed items in the Wave 1 data to covary – specifically items 12 and 13 (“There have been occasions when I have taken advantage of someone”; “I sometimes try to get even rather than forgive and forget”), and 10 and 14 (“I sometimes tell lies if I have to”; “I have said something bad about a friend behind his/her back”) – the fits indices improved (Exact fit χ^2^ = 844, *p* < 0.001; CFI = 0.63; TLI = 0.56; RMSEA = 0.077; SRMR = 0.078), but the exact and incremental fits still did not reach reference values. Applying the same model modification in Wave 2 data worsened all fit indices except for the exact fit. Since the prior model did not fit the data well, we conducted EFA on the Wave 1 dataset to explore the underlying structure of our data.

**Table 2:** Confirmatory Factor Analysis fit indices for original 16-item 2-factor scale.

	Exact fit test ($\chi _{{103}}^{2}$)	CFI	TLI	RMSEA	SRMR
Wave 1	994, *p* < 0.001	0.56	0.48	0.083	0.082
Wave 2	1354, *p* < 0.001	0.50	0.42	0.098	0.081
*Cutoff*	*p* > 0.05	*> 0.93*	*> 0.95*	*< 0.05*	*< 0.08*

CFI: comparative fit index, TLI: Tucker-Lewis Index, RMSEA: root mean square error of approximation, SRMR: standardised root mean square residual.

**Figure 1:**
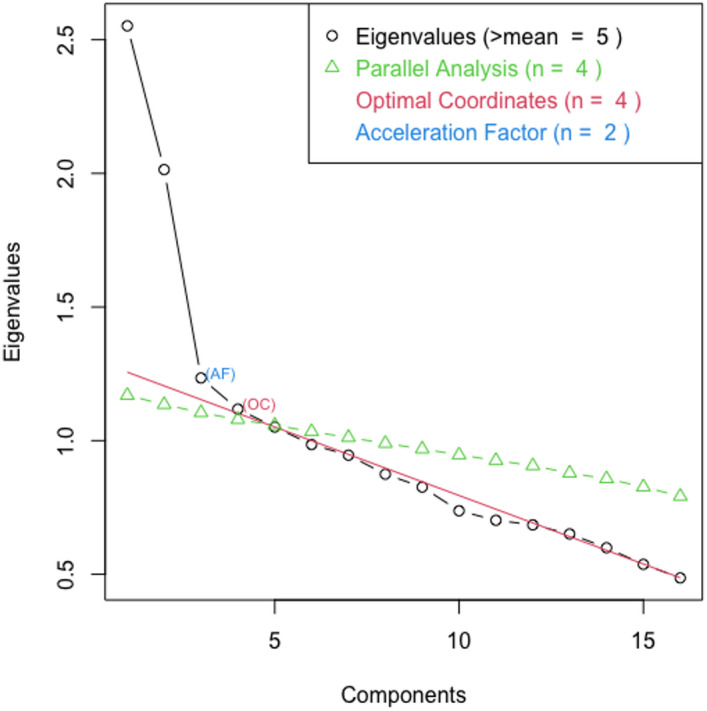
Scree plot for BIDR questions in Wave 1.

*Exploratory factor analysis.* The scree plot for Wave 1 data suggested a two-factor structure (Fig 1), as did Velicer’s MAP (the factor solution that minimizes the average squared partial correlations). Parallel analysis suggested retaining four factors, however, keeping more than two factors led to two-item factors. Given the inconsistency we chose to focus on the two-factor structure. We built this by making stepwise deletions of items with loadings of < 0.3 or cross loadings with differences < 0.1. In Wave 1 this removed five items, all from the original SDE subscale (Table 3). After dropping these items, the other loadings did not increase (Supplementary Table S3). The EFA loadings after item reduction proposed an 11-item, 2-factor scale. There was no clear pattern to the loading based on question content, but notably all reverse-coded items fit within one factor.

**Table 3:** Factor loadings after oblique rotation, ranked by loading on assigned factor.

	Factor 1	Factor 2
16 (IM) I never take things that don’t belong to me	**0.600**	0.07
15 (IM) When I hear people talking privately, I avoid listening	**0.544**	0.081
17 (IM) I don’t gossip about other people’s business	**0.526**	0.102
7 (SDE) I am a completely rational person	**0.516**	-0.112
11 (IM) I never cover up my mistakes	**0.425**	-0.104
14 (IM) I have said something bad about a friend behind his/her back*	0.009	**0.507**
12 (IM) There have been occasions when I have taken advantage of someone*	0.096	**0.464**
13 (IM) I sometimes try to get even rather than forgive and forget*	0.098	**0.420**
10 (IM) I sometimes tell lies if I have to*	0.154	**0.419**
6 (SDE) I sometimes lose out on things because can’t make up my mind*	-0.169	**0.385**
2 (SDE) I have not always been honest with myself*	0.132	**0.339**
9 (SDE) I have sometimes doubted my attractiveness to the other sex*	-0.280	0.334
5 (SDE) I never regret my decisions	0.211	-0.081
3 (SDE) I always know why I like things	0.160	-0.097
8 (SDE) I am very confident of my judgments	0.096	0.057
4 (SDE) It’s hard for me to shut off a disturbing thought*	-0.254	0.293

Bolded values are for loadings ≥ 0.3; Italicized values have a loading difference of < 0.1; *reverse coded items. IM and SDE refer to the factors each question was originally assigned to by BIDR; IM: Impression management; SDE: Self-deceptive enhancement.

We tested the new 11-item, 2-factor structure on Wave 2 data. Additionally, we included a third factor to the model containing all those items that loaded on neither or on both factors. For the 11-item scale, the two incremental fit indices improved, the absolute fit indices only partly. Overall, improvements were marginal and still did not meet recommended reference values (Table 4).

**Table 4:** Model fit indices for 16- and 11-item scale with 2 factors and 16-item scale with 3 factors.

Model	Exact fit test ($\chi _{{}}^{2}$)	CFI	TLI	RMSEA	SRMR
Original 16-item	1354 (103 df), *p* < 0.001	0.50	0.42	0.098	0.081
New 11-item	608 ( 43 df), *p* < 0.001	0.62	0.51	0.101	0.077
+ 3rd factor	1304 (101 df), *p* < 0.001	0.52	0.43	0.097	0.080
*Cutoff*	*p* > 0.05	*> 0.93*	*> 0.95*	*< 0.05*	*< 0.08*

CFA: confirmatory factor analysis, CFI: comparative fit index, TLI: Tucker-Lewis Index, RMSEA: root mean square error of approximation, SRMR: standardised root mean square residual, df: degrees of freedom.

*Measurement Invariance*. There was no significant difference by age in model fit between models M1 and M2, confirming factor loadings were age-invariant, and no difference between models M2 and M3, confirming scalar invariance also held. The significant difference between M1 and M2 for gender showed that metric invariance could not be assumed, i.e., factor loading patterns differed between male and female respondents. Between interviews conducted in French and Dioula, constraining the factor loadings to be equal (M2) led to a decline in model fit just above the common cutoff, tentatively suggesting metric invariance. Constraining intercepts worsened fit so that scalar invariance is not supported (Table 5).

**Table 5:** Measurement invariance across age groups (12–15 and 16–21), gender (female and male) and language (French and Dioula) for Wave 2.

	χ^2^ (df)	CFI	RMSEA	SRMR	Model compared	∆χ^2^ (∆df = 9)	∆CFI	∆RMSEA	∆SRMR
Age									
M1 configural	649 (86)	0.62	0.10	0.07					
M2 metric	656 (95)	0.62	0.10	0.07	M1	7.55	0.001	-0.005	0.001
M3 scalar	665 (104)	0.62	0.09	0.07	M2	8.50	0	-0.004	0
Gender									
M1 configural	766 (86)	0.59	0.11	0.08					
M2 metric	921 (95)	0.51	0.12	0.10	M1	156*	-0.09	0.005	0.015
Language									
M1 configural	705* (86)	0.57*	0.11	0.08*					
M2 metric	733 (95)	0.56	0.10	0.08	M1	27.9	-0.013	-0.004	0.001
M3 scalar	767 (104)	0.54	0.1*	0.09	M2	33.7	-0.017	-0.003	0.002

* *p* < 0.0001. CFI: comparative fit index, RMSEA: root mean square error of approximation, SRMR: standardised root mean square residual, df: degrees of freedom.

### Reliability

Internal consistency was low across waves and subscales for the original scale structure, and did not increase for the new scale applied to Wave 2 data (Table 6). Similarly, test-retest reliability was low across rounds, with 1263 complete observations allowing for different raters in each round. The interrater correlation coefficient ICC(A,1) was 0.06, as was the Pearson’s correlation coefficient, and the model variance explained (R^2^) when regressing Wave 2 values on Wave 1 ones was 0.004.

**Table 6:** BIDR internal consistency for original complete scale and original SDE and IM subscales for Wave 1 and 2 and the new 11-item scale for Wave 2.

	Original scale		New scale
	Wave 1	Wave 2	Wave 2
Cronbach’s α			
Complete	0.57	0.63	0.58
SDE/Subscale 1 for new scale	0.4	0.35	0.43
IM/Subscale 2 for new scale	0.63	0.57	0.52
McDonald’s ω_h_			
Complete	0.19	0.29	0.12
SDE/Subscale 1 for new scale	0.12	0.04	0.2
IM/Subscale 2 for new scale	0.33	0.12	0.28
McDonald’s ω_t_			
Complete	0.62	0.67	0.63
SDE/Subscale 1 for new scale	0.49	0.44	0.51
IM/Subscale 2 for new scale	0.68	0.63	0.61

IM: impression management, SDE: self-deceptive enhancement.

### Validity

In Wave 1 data, the 11-item BIDR scale showed positive convergent validity strongly with normative hygienic handwashing customs and weakly with life satisfaction, and negative convergent validity with reporting of alcohol consumption and past sexual violence; formal education was not significantly associated with the BIDR, mirroring mixed past evidence (Table 7). The limited association of BIDR with nutritional status and diet supported discriminant construct validity.

In Wave 2, normative handwashing customs and sexual violence continued to be significantly associated with the BIDR, as did the new variable of confidence in own judgement. However, life satisfaction had a reversed negative relationship with BIDR and alcohol use was no longer associated at all. For the discriminant constructs, both were negatively associated with BIDR in Wave 2, especially dietary diversity. Regression results for the original scale can be found in Supplementary Table S2.

**Table 7:** Linear regression analysis between predictor variables and newly identified 11-item scale and its two subscales.

	Scale	Wave 1			Wave 2		
		n (% missing)	Beta	95% CI	n (% missing)	Beta	95% CI
SLSS	Overall	1262 (2.3)	0.06	0.01, 0.12	1279 (0.9)	-0.11	-0.17, -0.06
	Subscale 1		-0.03	-0.09, 0.03		-0.01	-0.07, 0.04
	Subscale 2		0.14	0.08, 0.19		-0.17	-0.22, -0.11
Handwashing	Overall	1269 (1.7)	0.79	0.43, 1.15	1285 (0.5)	0.33	0.15, 0.51
	Subscale 1		1.23	0.88, 1.6		0.19	0.01, 0.37
	Subscale 2		-0.05	-0.42, 0.31		0.32	0.15, 0.50
Highest school level	Overall	1268 (1.8)	0.02	-0.05, 0.10	1284 (0.5)	-0.09	-0.14, -0.03
	Subscale 1		-0.02	-0.10, 0.06		-0.08	-0.14, -0.03
	Subscale 2		0.06	-0.02, 0.13		-0.05	-0.11, -0.00
Alcohol use ^c^	Overall	1266 (1.9)	-0.35	-0.61, -0.09	1268 (1.8)	-0.01	-0.19, 0.18
	Subscale 1		-0.33	-0.6, -0.07		-0.09	-0.27, 0.10
	Subscale 2		-0.21	-0.47, 0.05		0.08	-0.10, 0.26
Sexual violence ^c^	Overall	1208 (6.4)	-0.25	-0.33, -0.17	1253 (2.9)	-0.14	-0.21, -0.06
	Subscale 1		-0.23	-0.3, -0.15		-0.16	-0.23, -0.09
	Subscale 2		-0.16	-0.24, -0.08		-0.05	-0.12, 0.02
Nutritional status	Overall	1265 (2.0)	0.02	0.00, 0.05	1285 (0.5)	-0.03	-0.05, -0.01
	Subscale 1		0.02	0.00, 0.05		-0.02	-0.04, 0.00
	Subscale 2		0.01	-0.01, 0.03		-0.03	-0.05, -0.01
Diet	Overall	1264 (2.0)	0.03	-0.03, 0.08	1274 (1.3)	-0.13	-0.18, -0.08
	Subscale 1		0.01	-0.06, 0.07		-0.06	-0.11, -0.01
	Subscale 2		0.04	-0.03, 0.09		-0.13	-0.18, -0.09
Confidence ^b^	Overall				1275 (1.2)	0.12	0.07, 0.16
	Subscale 1					0.11	0.06, 0.16
	Subscale 2					0.07	0.03, 0.12

All analyses were adjusted for age and gender. ^b^ Wave 2 only. ^c^ Also adjusted for NVRC-arm. SLSS: students’ life satisfaction scale; CI: confidence interval.

## Discussion

We implemented the BIDR, a measure of socially desirable reporting developed in higher-income countries, in a low-education, highly resource-limited setting in northwestern Burkina Faso. Our evaluation did not confirm results previously reported for BIDR elsewhere, and scale evaluation tools suggested poor psychometric properties throughout. Factor loadings did not represent the scale’s original factor structure and loading patterns were inconsistent across waves. Our findings suggest that our BIDR implementation did not meaningfully measure a stable construct of socially desirable reporting in Burkina Faso. While there may be multiple explanations for this result, two lines of inquiry seem particularly worth considering.

One possible explanation for our inconsistent results is that SDR does not exist in our setting. This argument would suggest that SDR is a style rather than substance, since we would expect a trait to be present worldwide whereas if it was performance, it might not be present for some groups. Other evidence from across Africa suggesting the presence of SDR does not support this argument however^23,24,52^. Within SDR research generally, SDE is sometimes linked to substance and IM to style^25,53^, with a note that IM is more emphasized in collectivist societies^54^. However, given the poor properties for the entire scale, this seems of limited relevance here.

Alternatively, SDR may exist in our setting, but we failed to capture it, potentially due to translation, scale content or reverse-coding. The questionnaire was delivered in multiple languages used primarily orally in the setting. Although interviewers underwent thorough training including practicing translation of scale items from French (previously validated elsewhere) into Mooré or Dioula, this process inherently allows variation and error to occur. For the newly found factor structure, metric invariance – which accounts for loading patterns – was supported just above the cutoff, meaning that translation alone cannot explain the poor results. For the original BIDR factor structure, not even configural invariance was supported, suggesting the scale does not yield same structure.

Another explanation might be that the scale’s content was not representative of social desirability in our sample. Past implementation of the MCSDS in Ethiopia, Kenya, Mozambique and Uganda with a sample comparable to ours in age and education did not find such inconsistencies^24^, but differences may well exist across African regions.

Finally, the clustering of direct- and reverse-coded items in our EFA suggests that miscomprehension of the reverse-coding may have contributed to the poor scale properties in two ways. First, understanding of reverse-coding is subject to culture and language^55^, and reverse-coding is also known to induce miscomprehension in adolescent and low-formal-education samples^56^. Such low comprehension should generate lower within-group intercorrelations among reverse-coded items compared to directly-coded items. This is what we found, where loadings for the second Wave 1 EFA factor (predominantly reverse-coded items) are higher than those for the first factor (predominantly direct-coded items). Second, if respondents perceive reverse and directly worded items as unrelated they may answer them differently leading to all reverse-coded items loading onto a single factor; this phenomenon may be exacerbated by straight-line scoring within each group^56,57^. Albeit not discussed as such, an evaluation of the MCSDS in Singapore similarly found a novel 2-factor structure, with the second factor being represented only by reverse coded items^58^.

### Strengths and limitations

This analysis, and the study underlying it, had the strengths of longitudinal data on social desirability among a cohort of over 1000 adolescents. It also had several limitations. While we used a wide range of scale evaluation techniques, we were not able to conduct AVE and known groups comparison for construct validity, bifactor modelling for dimensionality testing^59^ and did not have access to personality data (e.g., Big Five) for convergent construct validity. We also did not have a second SDR scale for within-sample comparison and potential change of the underlying SDR construct in the intervening year – while unlikely – was not assessed^60^.

Given the primarily oral character of the local languages, we were also not able to conduct translation of the BIDR-16 in the typical written way, limiting the transparency of the translation process. While there is only weak evidence of measurement invariance by interview language, future work in settings without commonly used written language forms could benefit from careful evaluation of the translation complexity (and thus risk of bias) of each survey item, and thus the most appropriate method of translation – ranging from a simple, close translation from the original language (adoption), a culture-specific modification of some items (adaptation) or even a major revision of items or scale parts (assembly)^61^.

As discussed above, the psychometric properties (or lack thereof) of the BIDR-16 for our sample might not be generalisable across countries or populations. This limitation highlights the need to qualitatively explore what is socially desirable locally, potentially developing a culturally adjusted scale^61^. This scale development should preferably be community-led. There are very few published studies on formal co-design of scales specifically^62,63^. However, the application of co-design in other research settings suggest favourable outcomes including lower rates of screening failure and missing data and higher enrolment rates^64,65^. Developing a survey tool by engaging the community can ensure the relevance of questions for the target group^65^, and cause the power dynamics arising from “traditional research methods [to be] to some extent mitigated” and shifted^64^.

Unsurprisingly in a mobile age-group such as ours, loss-to-follow-up occurred, with 21% of baseline participants not re-interviewed. While non-consent at follow-up was rare (< 5% of non-participants), if consenting to re-interview is considered a sign of social desirability this may have affected sample representativeness. Lastly, the embedding of our scale within a longer interview may have led to participant (and interviewer) fatigue, reducing response validity.

## Conclusion

Our analysis highlights the well-known, but not always followed, dictum to validate an established tool in a new population. In this instance, some combination of oral translation, use of reverse-coding and age and formal education of the respondents appears to have led to a poor implementation of a potentially valid instrument. Future work to build locally applicable measures of social desirability bias in low-resource settings would benefit the field.

## Supplementary Information

Below is the link to the electronic supplementary material.

Supplementary Material 1

## Data Availability

Data are not publicly available due to consent not being given by participants for data to be shared openly, and due to the risk of deductive disclosure with sufficient local information given the inclusion of large proportions of age cohorts in the study villages. Anonymised data are available from ARISE study data controllers only following signature of a data use agreement restricting onward transmission. Anyone wishing to replicate the analyses presented, or conduct further collaborative analyses using ARISE (which are welcomed and considered based on a letter of intent), should contact Dr Guy Harling (g.harling@ucl.ac.uk) in the first instance.
