# Supplementary material for: Validating the Balanced Inventory of Desirable Reporting in a low literacy adolescent population in Burkina Faso
Source: Sci Rep. 2025 Nov 10;15:39311. doi: 10.1038/s41598-025-23145-1 (PMC12603020; doi:10.1038/s41598-025-23145-1)
Supplement: Supplementary file 1 — Supplementary Material 1 [file 41598_2025_23145_MOESM1_ESM.pdf]

Supplementary Table S1. Missing cases in predictor variables (n=1291 in both waves)

	Wave 1		Wave 2	
	Missing	%	Missing	%
Age	0	0	0	0
Gender	0	0	0	0
SLSS	13	1.0	6	0.5
Handwashing	0	0	0	0
Abuse	65	5.0	32	2.5
Alcohol	4	0.3	17	1.3
Current school enrolment	0	0	0	0
Highest level of education	1	0	1	0
Social norms experiment			10	0.8
Nutritional Status	4	0.3	0	0
Foods	7	0.5	11	0.9
BIDR IM	13	1.0	4	0.3

SLSS: students' life satisfaction scale, BIDR IM: Balanced inventory of desirable reporting, Impression management.

Supplementary Table S2. Linear regression analysis between predictor variables and original BIDR-scale and its two subscales

	Scale	Beta	95% CI	n	Beta	95% CI	n
SLSS total	IM	0.12	0.07, 0.18	1247	-0.12	-0.18, -0.07	1264
	SDE	0.08	0.03, 0.14	1247	0.03	-0.02, 0.08	1264
Handwashing	IM	-0.75	-1.11, -0.38	1250	0.04	-0.14, 0.22	1269
	SDE	-1.24	-1.62, -0.88	1250	0.06	-0.11, 0.23	1269
Schooling level	IM	0.07	0.00, 0.15	1249	0.22	-0.03, 0.08	1268
	SDE	0.01	-0.07, 0.08	1249	0.08	0.02, 0.13	1268
Alcohol [c]	IM	0.01	-0.26, 0.27	1248	-0.08	-0.11, 0.26	1252
	SDE	0.28	0.02, 0.56	1248	0.22	0.04, 0.39	1252
Sexual abuse [c]	IM	0.00	-0.07, 0.08	1192	0.07	0.00, 0.14	1238
	SDE	0.13	0.06, 0.22	1192	0.11	0.04, 0.17	1238
Nutritional Status	IM	-0.00	-0.02, 0.02	1246	-0.01	-0.03, 0.01	1269
	SDE	0.01	-0.02, 0.03	1246	0.01	-0.01, 0.03	1269
Diet	IM	0.06	-0.01, 0.12	1246	-0.05	-0.01, 0.00	1258
	SDE	-0.01	-0.07, 0.05	1246	0.07	0.03, 0.12	1258
Confidence [b]	IM				-0.09	-0.14, -0.04	1259
	SDE				0.05	0.00, 0.09	1259

All analyses adjusted for age and gender. [b] Wave 2 only. [c] Additionally adjusted for NVRC-arm.
SLSS: students' life satisfaction scale. CI: confidence interval.

Supplementary Table S3. EFA Wave 1 change in factor loading after stepwise item deletion according to loading

		16 item		- item 8		- item 3		- item 5		- item 4		- item 9	
Item		F1	F2	F1	F2	F1	F2	F1	F2	F1	F2	F2	F2
I have not always been honest with myself	2	0.13	**0.34**	0.13	**0.34**	0.13	**0.34**	0.14	**0.33**	0.14	**0.32**	0.14	**0.30**
I always know why I like things	3	0.16	-0.10	0.16	-0.10								
It's hard for me to shut off a disturbing thought	4	-0.25	0.29	-0.25	0.29	-0.25	0.29	-0.25	0.29				
I never regret my decisions	5	0.21	-0.08	0.20	-0.09	0.19	-0.08						
I sometimes lose out on things bc. can't make up my mind	6	-0.17	**0.39**	-0.17	**0.38**	-0.17	**0.38**	-0.16	**0.38**	-0.16	**0.36**	-0.16	**0.34**
I am a completely rational person	7	**0.52**	-0.11	**0.51**	-0.12	**0.51**	-0.12	**0.49**	-0.11	**0.47**	-0.10	**0.46**	-0.09
I am very confident of my judgments	8	0.10	0.06										
I have sometimes doubted my attractiveness to the other sex	9	-0.28	**0.33**	-0.28	**0.34**	-0.29	**0.34**	-0.29	**0.35**	-0.28	**0.32**		
I sometimes tell lies if I have to	10	0.15	**0.42**	0.15	**0.41**	0.16	**0.41**	0.15	**0.41**	0.12	**0.43**	0.10	**0.44**
I never cover up my mistakes	11	**0.42**	-0.10	**0.43**	-0.11	**0.42**	-0.10	**0.42**	-0.11	**0.42**	-0.10	0.40	-0.08
There have been occasions when I have taken advantage of so	12	0.10	**0.46**	0.10	**0.46**	0.09	**0.46**	0.09	**0.47**	0.05	**0.50**	0.02	**0.53**
I sometimes try to get even rather than forgive and forget	13	0.10	**0.42**	0.10	**0.42**	0.10	**0.43**	0.10	**0.43**	0.08	**0.43**	0.06	**0.43**
I have said something bad about a friend behind his/her back	14	0.01	**0.51**	0.01	**0.51**	0.01	**0.51**	0.02	0.50	-0.03	**0.54**	-0.05	**0.56**
When I hear people talking privately, I avoid listening	15	**0.54**	0.08	**0.54**	0.08	**0.54**	0.08	**0.53**	0.08	**0.54**	0.06	**0.57**	0.03
I never take things that don't belong to me	16	**0.6**	0.07	**0.61**	0.07	**0.61**	0.07	**0.62**	0.06	**0.64**	0.04	**0.64**	0.02
I don't gossip about other people's business	17	**0.53**	0.10	**0.53**	0.10	**0.54**	0.10	**0.55**	0.09	**0.57**	0.06	**0.59**	0.03

Loadings > 0.3 in bold.

Supplementary Table S4. Balanced inventory of desirable reporting – 16 item questions as used in the Nouna ARISE questionnaire

	English version	Version française
1	I have not always been honest with myself	Je n'ai pas toujours été honnête envers moi-même
2	I always know why I like things	Je sais toujours pourquoi j'aime quelque chose
3	It's hard for me to shut off a disturbing thought	Il m'est difficile de faire abstraction d'une pensée qui me trouble
4	I never regret my decisions	Je ne regrette jamais mes décisions
5	I sometimes lose out on things because I can't make up my mind soon enough	Je perds parfois de bonnes occasions parce que je prends trop de temps à me décider
6	I am a completely rational person	Je suis une personne complètement rationnelle
7	I am very confident of my judgments	J'ai énormément confiance en mon jugement
8	I have sometimes doubted my attractiveness to the other sex	J'ai parfois douté de mon attrait/attractivité à l'autre sexe
9	I sometimes tell lies if I have to	Parfois je mens, s'il le faut
10	I never cover up my mistakes	Je ne cherche jamais à dissimuler les erreurs que j'ai commises
11	There have been occasions when I have taken advantage of someone	Il m'est arrivé(e) de profiter de quelqu'un
12	I sometimes try to get even rather than forgive and forget	J'essaie parfois de me venger plutôt que de pardonner et d'oublier
13	I have said something bad about a friend behind his/her back	J'ai parlé en mal d'un(e) ami(e) dans son dos
14	When I hear people talking privately, I avoid listening	Lorsque je surprends une conversation privée, j'évite d'écouter
15	I never take things that don't belong to me	Je n'utilise jamais des choses qui ne m'appartiennent pas
16	I don't gossip about other people's business	Je ne fais pas de commérage au sujet des affaires des autres

Response options for each question ranged from 1 “not true” to 7 “very true” in English, and from 1 “pas vrai” to 7 “tres vrai” in French.

Supplementary Table S5. Students' Life Satisfaction Scale (SLSS) questions as used in the Nouna ARISE questionnaire

	English version	Version française
1	My life is going well	Ma vie se passe bien
2	I would like to change many things in my life	J'aimerais changer beaucoup de choses dans ma vie
3	My life is just right	Ma vie est juste comme il faut
4	I wish I had a different kind of life	J'aimerais avoir une vie différente
5	I have a good life	J'ai une vie agréable
6	I have what I want in life	J'ai ce que je veux dans la vie
7	My life is better than that of most other kids	Ma vie est meilleure que celle de la plupart des autres jeunes

Response options in English were: (1) strongly disagree; (2) moderately disagree; (3) mildly disagree; (4) mildly agree; (5) moderately agree; (6) strongly agree.

Response options in French were: (1) pas du tout d'accord; (2) plutôt en desaccord; (3) peu en desaccord; (4) peu d'accord; (5) plutôt d'accord; (6) tout à fait d'accord.
